# COVID-19 and Alzheimer’s Disease

**DOI:** 10.3390/brainsci11030305

**Published:** 2021-02-27

**Authors:** Marcello Ciaccio, Bruna Lo Sasso, Concetta Scazzone, Caterina Maria Gambino, Anna Maria Ciaccio, Giulia Bivona, Tommaso Piccoli, Rosaria Vincenza Giglio, Luisa Agnello

**Affiliations:** 1 Institute of Clinical Biochemistry, Clinical Molecular Medicine and Laboratory Medicine, Department of Biomedicine, Neurosciences and Advanced Diagnostics, University of Palermo, 90127 Palermo, Italy; bruna.losasso@unipa.it (B.L.S.); concetta.scazzone@unipa.it (C.S.); cmgambino@libero.it (C.M.G.); giulia.bivona@unipa.it (G.B.); rosaria.vincenza.giglio@alice.it (R.V.G.); luisa.agnello@unipa.it (L.A.); 2 Department of Laboratory Medicine, University Hospital “P. Giaccone”, 90127 Palermo, Italy; 3 Unit of Clinical Biochemistry, University of Palermo, 90127 Palermo, Italy; amciaccio21@gmail.com; 4 Unit of Neurology, Department of Biomedicine, Neurosciences and Advanced Diagnostics, University of Palermo, 90127 Palermo, Italy; tommaso.piccoli@unipa.it

**Keywords:** AD, biomarkers, SARS-CoV-2, neuroinflammation, neurodegenerative nisease, nervous system

## Abstract

The Severe Acute Respiratory Syndrome Coronavirus 2 (SARS-CoV-2) is a neurotropic virus with a high neuroinvasive potential. Indeed, more than one-third of patients develop neurological symptoms, including confusion, headache, and hypogeusia/ageusia. However, long-term neurological consequences have received little interest compared to respiratory, cardiovascular, and renal manifestations. Several mechanisms have been proposed to explain the potential SARS-CoV-2 neurological injury that could lead to the development of neurodegenerative diseases, including Alzheimer’s Disease (AD). A mutualistic relationship between AD and COVID-19 seems to exist. On the one hand, COVID-19 patients seem to be more prone to developing AD. On the other hand, AD patients could be more susceptible to severe COVID-19. In this review, we sought to provide an overview on the relationship between AD and COVID-19, focusing on the potential role of biomarkers, which could represent precious tool for early identification of COVID-19 patients at high risk of developing AD.

## 1. Introduction

The Severe Acute Respiratory Syndrome Coronavirus 2 (SARS-CoV-2) is the pathogen responsible for COVID-19 disease, which is characterized by a wide spectrum of symptoms, from fever and cough to multiple organ dysfunctions [1]. Additionally, SARS-CoV-2 can induce, directly or indirectly, several complications involving different organs [2,3]. Nowadays, the clinical course of the infection is unpredictable and characterized by high inter-individual variability. However, more than 80% of COVID-19 patients present ageusia or anosmia, which occurs early during the infection and represent pathognomonic features of the disease [4]. 

SARS-CoV-2, as well as all members of the human coronaviruses (CoVs) family, is an opportunistic pathogen of the central nervous system (CNS) [5]. The neurological signs and symptoms associated with SARS-CoV-2 infection, such as confusion, headache, hypogeusia/ageusia, hyposmia/anosmia, dizziness, epilepsy, acute cerebrovascular disease [4], are caused by the direct invasion of the virus into the CNS, and the subsequent interaction between SARS-CoV-2 spike protein and the angiotensin-converting enzyme 2 (ACE2) [6,7,8]. Post-mortem studies revealed the presence of both SARS-CoV-2 antigen and RNA in the brain tissue of COVID-19 patients [9].

ACE-2 expression is a key determinant of viral tropism and COVID-19 pathogenesis. In the brain, ACE-2 is expressed both on neurons and glial cells as well as on endothelial and arterial smooth muscle cells. ACE-2 is also expressed on the temporal lobe and hippocampus, which represent cerebral regions involved in the pathogenesis of Alzheimer’s Disease (AD) [6]. 

It has been hypothesized that SARS-CoV-2 could cause damage in the CNS by direct neurotoxicity or indirectly through the activation of the host immune response, which could lead to demyelination, neurodegeneration and cellular senescence. Thus, it could accelerate brain aging favoring the development of neurodegenerative diseases, including dementia [10]. However, after the acute recovery phase, the long-term consequences of SARS-CoV-2 infection on accelerated aging and age-related neurodegenerative disorders are actually unknown. Noteworthily, SARS-CoV-2 could potentially induce a worsening cognitive decline in AD patients.

**Figure 1:**
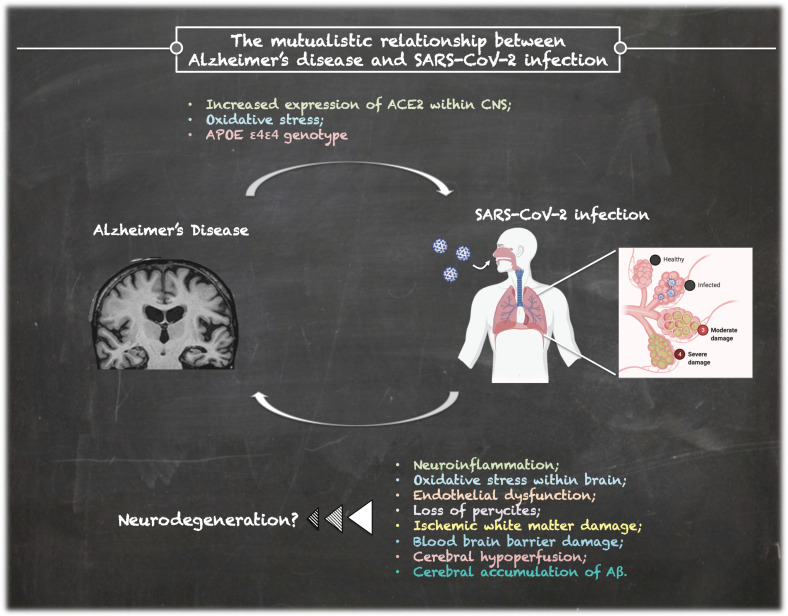
The complex relationship between Alzheimer’s Disease and SARS-CoV-2 infection.

On the other hand, dementia could represent an important risk factor for COVID-19 severity and mortality, as shown by preliminary reports [11,12]. Thus, a mutualistic relationship between SARS-CoV-2 infection and AD can be hypothesized. Figure 1 shows the possible association between AD and SARS-CoV-2 infection by summarizing the possible underlying mechanisms, which are described in the next paragraphs.

In this review, we sought to provide an overview on the relationship between AD and COVID-19, focusing on the potential role of biomarkers. This should represent a starting point for further investigations.

## 2. COVID-19 in Alzheimer’s Disease

AD represents the most common form of dementia worldwide [13]. The term dementia refers to a wide spectrum of disorders characterized by global, chronic and generally irreversible cognitive deterioration, leading to the progressive alteration of several functions such as memory, the ability to orient oneself, and alterations of the personality and behavior, which compromise the autonomy of the subject in the daily life [13,14]. The incidence of dementia is increasing in the general population. Indeed, the World Health Organization and Alzheimer Disease International Report of 2016 defined it as a global public health priority [15]. Patients with dementia are frail, dependent on caregivers for daily living activities and need the support of several services resources, such as physical exercise and physiotherapy [16]. Thus, the measures introduced by government authorities during the current COVID-19 pandemic, including confinement and isolation, may exacerbate the cognitive decline. Additionally, patients with AD and mild dementia may either be unwilling or unable to follow recommendations from public health authorities such as sanitize their hands, cover their mouth and nose when coughing, maintain physical distance from others, in part due to the severity of their short-term memory loss and overall cognitive impairment [17]. 

The brain of AD patients is characterized by amyloid plaque deposition and the presence of neurofibrillary tangles, which induce neuronal damage and synapse loss as well as oligodendroglia degeneration and myelin impairment [18].

Post-mortem studies showed that ACE-2 expression is increased in the brain of AD patients in comparison to controls [19]. Additionally, genome-wide association studies (GWAS) showed that the expression of ACE-2 gene is elevated in the brain tissue of AD patients with increased levels in severe forms [20]. Thus, enhanced ACE-2 expression could represent a risk factor for COVID-19 transmission in AD patients. It has been postulated a direct link between AD and ACE-2 expression mediated by oxidative stress. Specifically, aging leads to the imbalance in the redox state, characterized by the generation of excess reactive oxygen species (ROS) or the dysfunction of the antioxidant system, leading to oxidative stress [21]. AD patients show a significant extent of intracerebral oxidative damage associated with the abnormal marked accumulation of Aβ and the deposition of neurofibrillary tangles [21]. Interestingly, ACE2 inhibitors have recently been suggested as potential treatment for neurodegenerative diseases, including AD [22]. 

Noteworthily, AD and COVID-19 share several risk factors and comorbidities, such as age, gender, hypertension, diabetes and APOE ε4 expression. Such evidence could in part explain the increased prevalence of SARS-CoV-2 infection in AD patients. However, further studies are mandatory in order to clarify the pathophysiological mechanisms linking AD and COVID-19.

## 3. Patients with COVID-19 Could Develop AD?

Overall, CoVs can enter the CNS via different routes, including retrograde axonal transport via the olfactory and enteric neurons or infected lymphocytes, which cross the disrupted blood-brain barrier (BBB) [23].

Aging is characterized by a gradual loss of the BBB integrity [24]. Thus, the elderly could be more susceptible to neuroinvasion during SARS-CoV-2 infection.

SARS CoV-2 infects the olfactory neurons and, through the neuro-epithelium of the olfactory mucosa, reaches the olfactory bulb in the hypothalamus [5,25]. The presence of SARS CoV-2 in the olfactory bulb leads to the activation of non-neuronal cells, such as mast cells, microglia, astrocytes, as well as to the tissue release of pro-inflammatory cytokines. SARS-CoV-2 uses the phospholipids of the infected cells to build its own envelope. The consequence is that the cells, in particular the innate immune cells, lose precursors for the synthesis of the autacoid local injury antagonist amides (ALIamides), which have a pivotal role for controlling the excessive reactivity [26]. Consequently, the resulting neuroinflammation could become uncontrollable, especially in the elderly, which have a less efficient immune system response [27,28]. Neuroinflammation, associated with intense oxidative stress, could induce neurodegeneration, potentially favoring the development of neurodegenerative diseases, such as AD [25,29]. COVID-19 patients with advanced age and comorbidities with an inflammatory basis, such as diabetes, atherosclerosis and sub-clinical dementia, could be at increased risk of developing AD. 

Several pathological mechanisms seem to be involved in the potential increased risk of developing AD in COVID-19 patients. 

A growing body of evidence suggested a role for neuroinflammation. Systemic inflammation induces the activation of microglia and astrocytes, which in turn secrete pro-inflammatory cytokines, including IL-1β, IL-6, IL-12, TNF-α. Such biomarkers could be involved in the synaptic dysfunction, inducing neurodegeneration, which could potentially lead to AD [30]. 

Hypoxic alterations and demyelinating lesions have been described in COVID-19 patients [31,32,33]. Neuroradiological studies showed alterations of functional brain integrity, especially in the hippocampus, in recovered COVID-19 patients at 3-month follow-up. The hippocampus is an area particularly vulnerable to respiratory viral infections, as shown in experimental studies [34]. Hippocampal atrophy is associated with cognitive decline and represents a common characteristic of AD patients [35,36]. Additionally, the altered BBB could allow the infiltration of immune cells, which may contribute to cognitive decline and dementia in COVID-19 patients. Moreover, endothelial dysfunction, which is a pathognomonic characteristic of COVID-19, and loss of pericytes could impair the clearance of cerebral metabolites, including Aβ peptides. The excess and accumulation of Aβ protein in senile plaques, especially in the hippocampus, represent the main pathophysiological mechanism underlying the AD. Some authors showed that severe COVID-19 presents ischemic white matter damage due to the reduced perfusion secondary to hypercoagulability and disseminated intravascular coagulation (DIC), which are common features of severe COVID-19. Neuroimaging and experimental studies showed that ischemic white matter damage occurs at a very early stage of AD, accelerates the progression of the disease and contributes to cognitive decline [37,38]. Moreover, cerebral hypoperfusion can increase the phosphorylation rate of tau [39]. 

In severe COVID-19, the systemic inflammation characterized by the so-called “cytokine storm” leads to the disruption of the blood–brain barrier and neural and glial cell damage that could be involved in long-term sequelae. Systemic inflammation is recognized as a pathophysiological mechanism underlying AD [40]. Also, pro-inflammatory cytokines alter the capacity of the microglial cells to phagocyte b-amyloid, promoting the accumulation of amyloid plaques [41]. The virus-induced systemic inflammatory storm, associated with a massive release of mediators able to access the CNS due to the increased permeability of the blood-brain barrier, could amplify neuroinflammation and contribute to the neurodegeneration process [42].

Another interesting piece of evidence suggests that the potential increase of AD risk in COVID-19 patients could be related to Aβ, which can act as an antimicrobial peptide. Thus, it could be postulated that the SARS-CoV-2 neuroinvasion could promote Aβ generation, as part of the immune response, and the b-amyloid cascade leading to b-amyloid deposition [43]. However, this is only a hypothesis that must be proved. 

McLoughlin et al. showed that COVID-19 hospitalized patients who developed delirium during their hospitalization, after 1-month discharge had lower cognitive scores [44]. However, the difficulty in performing neuropsychological assessment leads to a poor understanding of the neurological impact of SARS-CoV-2 infection. Overall, ARDS is associated with a high prevalence of long-term cognitive impairment in critically ill patients [45]. Specifically, mechanical ventilation, which is a standard therapy to maintain adequate gas exchange during ARDS, also in severe COVID-19 patients, could contribute to long-term cognitive impairment [46,47,48,49]. Experimental studies showed that short-term mechanical ventilation triggers the neuropathology of AD by promoting cerebral accumulation of the Aβ peptide, systemic and neurologic inflammation, and blood–brain barrier dysfunction [50]. 

The long-term complications of COVID-19 would be expected in the next 10–15 years. Nowadays, it is not possible to assess them because the pandemic started last year. However, in the future, it will be pivotal to evaluate the risk of long-term COVID-19 neurological sequelae, especially in the elderly and patients who developed severe forms. 

The potential mechanisms involved in cognitive impairment in COVID-19 patients can be summarized as follows: (i) direct SARS-CoV-2 infection in the CNS; (ii) systemic hyper inflammatory response to SARS-CoV-2; (iii) cerebrovascular ischemia due to endothelial dysfunction; (iv) severe coagulopathy; v) mechanical ventilation due to ARDS or severe disease; (vi) peripheral organ dysfunction. 

Table 1 summarizes the potential mechanisms linking SARS-CoV-2 infection and the development of AD.

## 4. Biomarkers of Cognitive Decline in COVID-19 Patients

### 4.1. Neuronal Injury 

Biomarkers of neurodegeneration in the cerebrospinal fluid (CSF), such as tau proteins, neurofilament light chain protein (NfL), and glial fibrillary acidic protein (GFAp), are increased in COVID-19 patients and associated both with neurological symptoms and disease severity [53,54,55,56,57].

T-tau is a biomarker of neuronal death. Its levels are increased in several neurodegenerative diseases, including AD. Specifically, the biochemical diagnosis of AD relies on the detection of a CSF biomarker profile characterized by the decrease of amyloid beta 1-42 (Aβ 1-42), the ratio Aβ 1-42/1-40 and the increase of t-Tau and p-Tau levels [13]. Some Authors found that COVID-19 patients have an increase of CSF t-Tau levels suggesting the presence of neuronal damage. However, to date, levels of amyloid beta have never been investigated in such patients. 

Among intermediate filaments expressed in cerebral cells, GFAp and Neurofilmanents have been evaluated in COVID-19 patients. 

GFAP is highly expressed in astrocytes and represents a biomarker of astrocytic activation/injury [58]. AD is characterized by amyloid plaques surrounded by reactive astrocytes, which show an increased expression of intermediate filaments, including GFAP [58]. To date, only two studies evaluated the role of GFAp in COVID-19 patients [53,54]. The authors showed that severe COVID-19 patients had higher plasma concentrations of GFAp than controls. 

Neurofilaments are cytoskeletal proteins of neurons, particularly abundant in axons. Neurofilaments comprise three subunits: neurofilament light chain (NF-L), neurofilament medium (NF-M) and neurofilament heavy (NF-H). Among these, NF-Ls are the most abundant. 

Following axonal damage, NFs are released into CSF. Thus, they represent a biomarker of axonal damage and neuronal death. CSF NFs levels are increased in several neurological disorders, including AD [59]. Increased levels of serum and CSF NF-L have been found in severe COVID-19 patients [53,54,55,56,57,60]. 

Only one study evaluated t-Tau in COVID-19 patients and reported increased levels of t-Tau in severe cases. 

To date, a few authors evaluated the CSF biochemical profile of COVID-19 patients due to the difficulty of obtaining such biological fluid. However, preliminary literature evidence raises awareness for potential long-term neurologic sequelae following COVID-19. Although severe COVID-19 patients have CSF biochemical alterations indicative of neuronal and axonal damage, it is not possible to draw definitive conclusions on the cognitive impairment. Longitudinal studies are required to evaluate the potential neurological sequelae and the risk of developing AD. 

### 4.2. Genetic Variants

The most important known predisposing risk factor for AD is the polymorphism APOE ε4, with the ε4ε4 (homozygous) genotype being associated with a 14-fold increase in AD risk. Specifically, APOE ε4 is correlated with low cerebral blood flow and subcortical ischaemic white matter damage, as well as neuroinflammation in AD patients [51]. Kuo et al. showed that individuals carrying APOE ε4 in homozygous had a higher prevalence of SARS-CoV-2 infection. Additionally, APOE ε4ε4 allele was associated with an increased risk of developing severe COVID-19, independently of dementia, and other comorbidities, including cardiovascular disease, and type-2 diabetes [52]. Thus, APOE ε4 represents a common risk factor for AD and SARS-CoV-2 infection. APOE ε4 could promote vulnerability to viral infection and neurodegeneration. Thus, it can be postulated that the SARS-CoV-2 infection could be a promoting factor for neurodegeneration in individuals with susceptible genetic variants [52].

However, the relationships between APOE ε4, COVID-19, and AD must be elucidated. 

### 4.3. Inflammatory Biomarkers

Some inflammatory biomarkers, including IL-6, IL-1, and galectin-3 (Gal-3), have been proposed as a link between COVID-19 and AD. 

IL-6 represents one of the most studied cytokines in COVID-19. Circulating increased levels of IL-6 are associated with a high risk of developing severe COVID-19 and mortality. Accordingly, it represents a reliable prognostic biomarker in SARS-CoV-2 infection [61]. IL-6 is also a prognostic biomarker of AD. Indeed, its increased levels are associated with the progression of the disease and worse cognitive performance [62]. Thus, IL-6 represents a common biomarker for COVID-19 and AD. 

IL-6 exerts its biological effects by the interaction with IL-6R, which can be expressed on the membrane of immune, epithelial and liver cells or it can be present in soluble form. The latter represents an agonist of IL-6. The complex IL-6/IL-6R can activate intracellular pathways involved in the immunoinflammatory response [62,63]. 

Alterations in IL-6 and IL-6R genes could be involved in the onset and progression of several diseases, including infectious diseases, such as COVID-19, and neurodegenerative diseases, such as AD [64,65,66]. The “Disease and Function analysis” performed by Strafella et al. showed that IL-6 and IL-6R could be involved in neuroinflammation, synaptic damage, microglia activation and cognitive impairment in AD pathogenesis [63]. 

Similar to IL-6, IL-1 represents a prognostic biomarker of SARS-CoV-2 infection, with increased levels associated with worse prognosis [67,68]. IL-1 is a pro-inflammatory cytokine produced by several cell types, including glia and neurons. IL-1 levels have been found to increase in the brain of AD [69]. In vitro studies reported that IL-1 could induce neuronal death by the direct effect on neurons or indirectly by glial production of neurotoxic substances. Additionally, IL-1 is involved in the physiological regulation of hippocampal plasticity and memory processes. Literature evidence showed that alterations of IL-1 levels, both positively (increase) and negatively (decrease), are associated with impaired memory functioning. Thus, the increased levels of IL-1 found in COVID-19 patients could enhance cognitive decline, leading to the development of AD [70]. 

Gal-3 is a carbohydrate-binding protein belonging to the family of lectins. It has pleiotropic functions, with a key role in several physiological and pathological processes, including inflammation and fibrosis [71,72,73]. Increased levels of Gal-3 have been found in severe COVID-19 patients. It has been postulated that Gal-3 promotes COVID-19 progression by supporting the hyper-inflammation reaction and lung fibrosis, which is associated with the acute phase of diffuse alveolar damage, edema, and hypoxia [74]. Increased levels of Gal-3 have also been described in the serum of AD patients [75]. Studies on AD animal models showed that Gal-3 could be involved in the Aβ aggregation and amyloid plaque formation [76]. Thus, it can be hypothesised that increased levels of Gal-3 in COVID-19 patients could also be involved in the damage leading to the development of AD. However, further studies are mandatory to confirm such a hypothesis. 

## 5. Conclusions

Nowadays, the question “Can SARS-CoV-2 infection increase the risk for development of Alzheimer’s Disease?” actually remains unanswered. There is an urgent need for prospective studies to address such question. 

Neurological sequelae, including the cognitive impairment leading to AD, could represent an important complication of COVID-19. Further detailed clinical, laboratory, and neuropathological studies will help to elucidate the underlying pathophysiological mechanisms of the COVID-19 neurological complications. A longitudinal follow-up of COVID-19 patients, especially older adults and severe cases, is required to detect the potential long-term neurological consequences of SARS-CoV-2 infection. In such scenario, biomarkers represent reliable tools for early monitoring of COVID-19 patients and early detection of those at high risk of developing neurological sequelae, such as AD. Currently, there is still little literature evidence to draw definitive conclusions. However, an important relationship between AD and COVID-19 seems to exist. 

## Figures and Tables

**Table 1:** Potential mechanisms involved in Alzheimer’s Disease (AD) risk in COVID-19 patients.

Pathway	Mechanisms	References
Aβ deposition	Aβ is an antimicrobial peptide produced in response to neural infection as part of the innate immune innate response	[18,21,43]
APOEε4	APOEε4 represents a risk factor for both COVID-19 and AD; APOEε4 enhance the disruption of BBB; APOEε4 has an important role in neuroinflammation, which contributes to the pathogenic mechanism underlying AD.	[51,52]
Neuroinflammation	ACE-2 is expressed in the cells of the CNS; SARS-CoV-2 can infect cells within CNS; Pro-inflammatory cytokines can enter the CNS by crossing the altered BBB; Inflammatory response within CNS can alter cells within CNS leading to cognitive decline.	[25,27,28,29,42]
Microglia activation	SARS-CoV-2 infection can induce microglial activation leading to neuronal loss; Microglia activation promotes oxidative stress within brain; Increased NO levels are neurotoxic and promote AD development.	[30,41]

ACE-2: Angiotensin Converting Enzyme 2; AD: Alzheimer’s Disease; APOE: Apolipoprotein E; BBB: Blood Brain Barrier; COVID-19: Coronavirus Disease 2019; CSN: Central Nervous System; NO: Nitric Oxide; SARS-CoV-2: Severe Acute Respiratory Syndrome Coronavirus 2.
